# Elucidating the Anticancer Mechanisms of Cinnamoyl Sulfonamide Hydroxamate: Insights From DNA Content Analysis and Gene Expression Profiling in Squamous Cell Carcinoma

**DOI:** 10.7759/cureus.57236

**Published:** 2024-03-30

**Authors:** Eapen Cherian, Manoj Goyal, Neeti Mittal, Susan Mathews, Muhammad Sagir

**Affiliations:** 1 Oral and Maxillofacial Pathology, Travancore Dental College Medicity, Kollam, IND; 2 Oral and Maxillofacial Surgery, Santosh Deemed to Be University, Ghaziabad, IND; 3 Pediatric Dentistry, Santosh Deemed to Be University, Ghaziabad, IND; 4 Prosthodontics, Travancore Dental College, Kollam, IND; 5 Conservative Dentistry and Endodontics, Royal Dental College Medicity, Pallakad, IND

**Keywords:** toluidine, anisidine, molecular mechanisms, apoptosis, cell cycle arrest, cinnamoyl sulfonamide hydroxamate derivatives, oral cancer

## Abstract

Background: Oral cancer is a major public health concern worldwide, with oral squamous cell carcinoma (OSCC) being one of its most common subtypes. Despite advances in diagnosis and management of this disease, there remains a need to develop new therapeutic approaches for better outcomes.

Objective: This study aimed to investigate the molecular mechanisms through which cinnamoyl sulfonamide hydroxamate derivatives exert their anticancer effects on OSCC.

Materials and methods: The derivatives were synthesized via multi-step processes and then characterized at the molecular level. Flow cytometry assay for DNA content and cell cycle distribution, anisidine/toluidine double staining for apoptosis detection, as well as reverse transcription polymerase chain reaction (RT-PCR) gene expression analysis, were performed on OSCC cell lines exposed to cinnamoyl sulfonamide hydroxamate derivatives.

Results: Flow cytometry unveiled remarkable changes in the distribution of cells throughout the OSCC cell line upon treatment with cinnamoyl sulfonamide hydroxamate derivatives. Consequently, it led to a noticeable decrease in cells at the G0/G1 phase, together with an increase at the S phase, thereby indicating a retardation at various points of the cycle. In addition, apoptotic morphological alterations have been observed by anisidine/toluidine double staining after some treatments with the compounds. RT-PCR analysis showed a marked increase in p21 gene expression levels, further supporting the compounds' ability to induce cell cycle arrest and apoptosis.

Conclusion: The research highlighted the potential of cinnamoyl sulfonamide hydroxamate derivatives as candidates for oral cancer, particularly OSCC treatment, shedding light on their operation at the molecular level and paving the way for the development of targeted therapies that could aid in the cure of oral cancer.

## Introduction

The escalating burden of cancer remains a paramount concern globally, with oral cancer constituting a significant component of this challenge [1,2]. Oral cancer encompasses a heterogeneous group of malignancies affecting various oral cavity sites and ranks among the top three cancers in India, representing a substantial public health issue [3,4]. Despite advancements in diagnostic and therapeutic modalities, oral cancer continues to exert a considerable toll, underscoring the imperative for novel therapeutic approaches [5].

The development of oral squamous cell carcinoma (OSCC) involves diverse genetic alterations that disrupt normal cellular homeostasis through multiple mechanisms. Oncogenes and tumor suppressor genes get out of control, causing abnormal signaling pathways that boost cell proliferation, weaken cell adhesion, and enhance invasion properties [6]. Consequently, OSCC tends to manifest aggressive behavior by invading surrounding tissues and spreading to distant sites; therefore, it has high mortality rates due to morbidity associated with its progression [7].

Traditional methods of detecting oral cancer have been based on clinical examination followed by biopsy and histopathological analysis. Although these techniques remain indispensable, early molecular diagnosis is yet fundamental for achieving optimal disease progression [7]. Furthermore, since there are multiple treatment options for oral cancer like chemotherapy, surgery, radiotherapy, targeted therapy, and immunotherapy, personalized approaches in line with individual patient characteristics and the stage of the disease are needed [8].

In this context, natural products have received substantial attention as potential anticancer agents [9]. Cinnamic acid, found in species like *Cinnamomum cassia*, has diverse pharmacological actions like antioxidant, antimicrobial, anti-inflammatory, and also anticancer properties [10]. For instance, cinnamoyl sulfonamide hydroxamate derivatives of cinnamic acid have shown excellent performance as anticancer agents by inhibiting enzymes such as histone deacetylase (HDAC) involved in cancer cell growth and survival [11].

The present study seeks to elucidate the mechanistic underpinnings of the anticancer activity of cinnamoyl sulfonamide hydroxamate derivatives against OSCC. By investigating their effects on key cellular processes such as apoptosis, cell cycle regulation, and gene expression, this research aims to provide insights into their therapeutic potential and pave the way for the development of novel treatment strategies for oral cancer.

## Materials and methods

The collaborative research project involved contributions from multiple stakeholders, each fulfilling specific roles. For instance, Caritas College of Pharmacy in Kottayam was responsible for the preparation and analysis of materials, while Biogenics in Trivandrum executed in vitro cell culture methods. The research progressed through several stages subsequent to obtaining ethical approval from Santosh University, Ghaziabad, India, which was granted under reference number 856/PO/Re/S/04/CPSEA. Details regarding the synthesis and molecular characterization, as well as the outcomes of antioxidant and cytotoxicity studies, have been previously documented [12].

The synthesis of cinnamoyl hydroxamate derivatives entailed a series of three principal steps.

To synthesize (E)-3-(4-(chlorosulfamoyl)phenyl)acrylic acid, cinnamic acid was mixed with chlorosulfonic acid and reacted at 35°C for four hours. The reaction progress was tracked using thin-layer chromatography (TLC) plates. The resulting yellow precipitate (anhydrous calcium chloride (CaCl_2_)) was filtered, washed, and shade-dried, yielding the desired product.

For the synthesis of (E)-3-(4-(N-(phenyl bromo) or (phenyl nitro) sulfamoyl)phenyl)acrylic acid, bromoaniline or nitroaniline was added to (E)-3-(4-(chlorosulfamoyl)phenyl)acrylic acid in water while maintaining pH 8 with sodium bicarbonate (NaHCO_3_). The reaction mixture was stirred at 35°C for four hours, then the pH was adjusted to 2 with hydrochloric acid (HCl). The resulting white precipitate was washed and dried, then recrystallized using ethyl acetate.

To synthesize (E)-N-hydroxy-3-(4-(N-(phenyl bromo) or (phenyl nitro) sulfamoyl)phenyl)acrylamide, compound 3 was reacted with ethyl chloroformate and N-methylmorpholine in dichloromethane at 35°C for five hours. The transformation of acrylic acid to acid chloride was confirmed using TLC. The addition of hydroxylamine solution in tetrahydrofuran (THF) completed the transformation to the corresponding hydroxamate derivative. Purification through column chromatography yielded two compounds (3a, 3b) for further analysis, as described in subsequent sections.

Analysis of DNA content and cell cycle distribution using flow cytometry

Oral cavity squamous cell carcinoma (OECM 1), unique human head and neck squamous cell carcinoma (UM-SCC 6), and human tongue squamous carcinoma (HSC-3) cell lines obtained from the oral cavity of female Swiss albino mice were utilized. After harvesting, the cells were treated with LC50 concentrations of the synthesized compounds (3a: 79.63 µg/mL; 3b: 160.05 µg/mL) for 24 hours. Following treatment, cells were fixed with ice-cold 70% ethanol and stained with propidium iodide and RNase solution in the dark for 30 minutes. Subsequently, flow cytometry analysis was conducted, gating based on untreated control cells, to determine DNA content and cell cycle distribution (using Abcam, Waltham, Massachusetts). This comprehensive analysis provided valuable insights into the effects of the synthesized compounds on cell proliferation and viability.

Determination of apoptosis by anisidine and toluidine double staining

Apoptosis determination was carried out using anisidine and toluidine double staining, a method enabling the morphological identification of necrotic and apoptotic cells through the utilization of DNA-binding dyes. Cultured OECM 1, UM-SCC 6, and HSC-3 cell lines were treated with LC50 concentrations of the synthesized compounds (3a: 79.63 µg/mL; 3b: 160.05 µg/mL) for 24 hours. Subsequently, the cells were rinsed with PBS and stained with Anisidine and Toluidine solutions. After a 10-minute incubation at room temperature, stained cells were examined under a fluorescence microscope equipped with a blue filter, allowing for the differentiation of various cell states based on fluorescence emission patterns. This staining technique facilitated the accurate assessment of apoptosis induction by the synthesized compounds.

Gene expression by real-time reverse transcriptase-polymerase chain reaction (RT-PCR): p21 gene expression

The total RNA isolation was carried out using the TRIzol method as per the manufacturer's instructions. Following the treatment of cells in a six-well plate with the LC50 concentration of the sample (3a: 79.63 µg/mL) for 24 hours, RNA extraction was performed. Briefly, chloroform was added to the sample, vigorously shaken, and then centrifuged to separate the aqueous layer. Isopropanol was added to precipitate RNA, followed by centrifugation to obtain the RNA pellet. After ethanol washing and centrifugation, the RNA pellet was air-dried and suspended in TE buffer. Subsequently, cDNA synthesis was carried out using the Thermo Scientific Verso cDNA Synthesis kit (Thermo Scientific, Waltham, Massachusetts). Gel electrophoresis of the synthesized cDNA was performed on a 1.5% agarose gel, followed by visualization using a gel documentation system (E gel imager, Invitrogen, Carlsbad, California). This method effectively separates and visualizes DNA fragments based on their charge and size when exposed to an electric field.

## Results

Analysis of DNA content and cell cycle distribution using flow cytometry

Anisidine staining resulted in a significant decrease in cells in the G0/G1 phase (%Gated: 34.0) compared to the control (%Gated: 70.4), with a notable increase in cells in the S phase (%Gated: 38.5). Toluidine staining also led to alterations in cell cycle phases, with a decrease in G0/G1 phase cells (%Gated: 53.1) and an increase in S phase cells (%Gated: 18.0) compared to the control. Chi-square statistics indicated significant differences in cell cycle distribution between the control and stained groups for G0/G1 and S phases (p < 0.00001), while no significant difference was observed for the G2/M phase (p = 0.302817) (Table 1).

**Table 1:** Cell cycle distribution analysis and chi-square statistics indicated significant differences in cell cycle distribution between the control and stained groups CV: coefficient of variation.

Treatment	Cell Cycle	G0/G1 (%)	S (%)	G2/M (%)
Control (unstained)	%Gated	70.4	14.8	10.9
	Mean	2636.1	4029.78	5277.1
	%CV	17.2	8.5	8.3
Anisidine (stained)	%Gated	34.0	38.5	12.2
	Mean	3049.9	3829.9	5586.3
	%CV	14.7	6.8	9.3
Toluidine (stained)	%Gated	53.1	18.0	14.5
	Mean	2769.7	3907.7	5461
	%CV	18.6	8.4	9.3
Chi-square statistics		19.09664	287.29061	4.8521
P value		<0.00001	<0.00001	0.302817

One-way ANOVA analysis revealed significant differences in mean values among the study groups (p < 0.05) (Table 2).

**Table 2:** One-way ANOVA analysis performed to determine the association between the means of the study groups The f-ratio value is 0.38203. The p-value is 0.0486554. The result is significant at p < 0.05.

Summary of Data	Control	Anisidine	Toluidine	Total
N	9	9	9	27
∑X	8663.7	11862.38	16388.9	36914.98
Mean	962.6333	1318.0422	1820.9889	1367.221
∑X^2^	23931940.97	46179594.49	88877772.87	158989308.3
Std. Dev.	1396.0648	1953.9856	2716.4722	2042.9838
Result details				
Source	SS	Df	MS	
Between groups	3348135.263	2	1674067.631	F = 0.038203
Within-groups	105170219.4	24	4382092.476	
Total	108518354.7	26		

**Figure 1:**
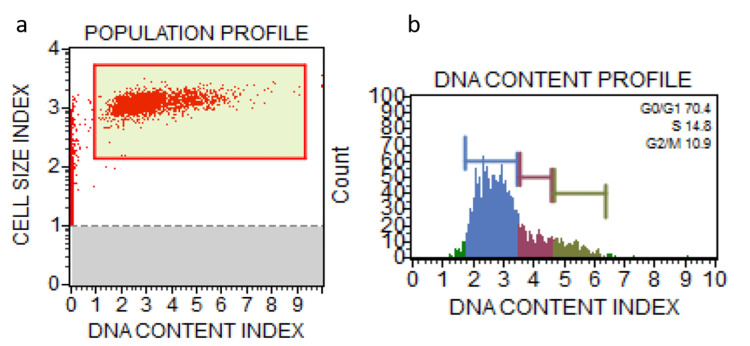
Population profile of untreated control cells (a) and DNA Content profile of untreated control cells (b) Red: G1 phase, Blue: G0/G1 phase, Green: G2M phase.

**Figure 2:**
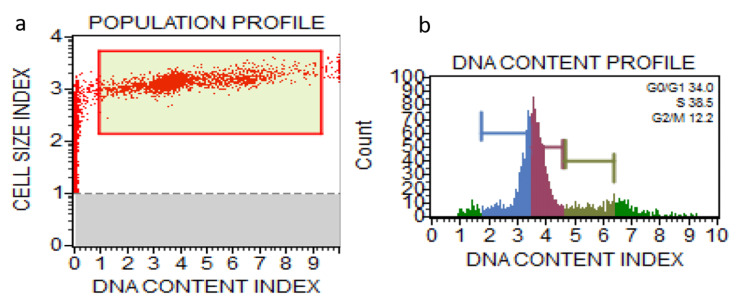
Population profile of cells treated with anisidine (a) and DNA content profile of cells treated with anisidine (b) Red: G1 phase, Blue: G0/G1 phase, Green: G2M phase, Purple; S phase.

**Figure 3:**
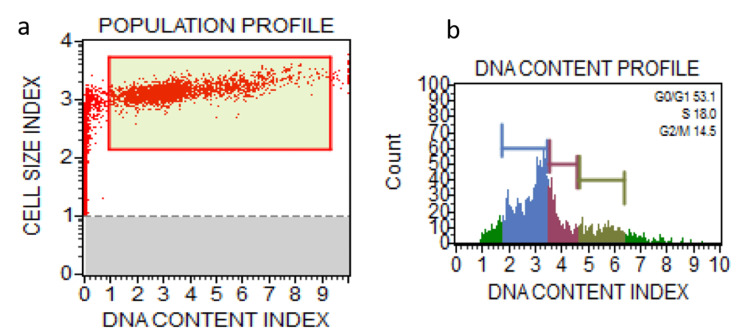
Population profile of cells treated with toluidine (a) and DNA content profile of cells treated with toluidine (b) Red; G1 phase, Blue: G0/G1 phase, Green: G2M phase, Purple: S phase.

Post hoc Tukey honestly significant difference (HSD) analysis pairwise comparisons showed significant differences between the control and anisidine groups (p = 0.043120) (Table 3 and Figures 1-3).

**Table 3:** Table showing the post hoc Tukey honestly significant difference (HSD) (beta) analysis *The result is significant at p < 0.05. T1: control; T2: anisidine; T3: toluidine.

Pairwise Comparisons	Mean Difference	HSD.05 = 2464.3553	Q.05 = 3.5317
		HSD.01 = 3171.8360	Q.01 = 4.5456
T1:T2	M1 = 962.63	355.41	Q = 0.41 (p = 0.043120)*
	M2 = 1318.04		
T1:T3	M1 = 962.63	858.36	Q = 1.23 (p = 0.66406)
	M3 = 1820.99		
T2:T3	M2 = 1318.04	502.95	Q = 0.72 (p = 0.86738)
	M3 = 1820.99		

Gene expression by RT-PCR: p21 gene expression 

RT-PCR analysis of p21 gene expression was performed to elucidate the molecular mechanism of cell cycle arrest induced by compounds 3a and 3b. Treatment with the compounds led to increased p21 expression, indicative of DNA damage-induced cell cycle arrest. Analysis of p21 expression levels across different cell lines revealed variations compared to the control. OECM-1 exhibited an increase in p21 expression (293602) compared to the control (258749). Similarly, UM-SCC 6 and HSC-3 cell lines also showed elevated p21 expression levels (319852 and 261198, respectively) (Table 4).

**Table 4:** p21 gene expression by reverse transcription polymerase chain reaction (RT-PCR)

Cell Line	p21 Expression (Ct value)
Control	258749
OECM-1	293602
UM-SCC 6	319852
HSC-3	261198

## Discussion

The reason underlying this study arises from the increasing interest in exploring the therapeutic potential of cinnamic acid derivatives, particularly for cancer therapy [11,12]. Earlier evidential studies have emphasized that these derivative series act as anticancer agents through various mechanisms, such as HDAC inhibition and the triggering of apoptotic pathways, providing a strong foundation for further work [10,13-15]. Moreover, cinnamic acid derivatives exhibit antioxidant activity and reduction potential, suggesting extended therapeutic applications beyond their use in cancer treatment. However, despite these promising findings, there is a need for extensive research regarding the efficacy and molecular mechanisms of action of specific compounds like cinnamyl sulfonamide hydroxamate compounds (derivatives 3a and 3b) [12]. Thus, this work intends to contribute to the existing knowledge on the anticancer activity, antioxidant properties, cytotoxic effects, and molecular mechanisms of cinnamyl sulfonamide hydroxamate compounds (derivatives 3a and 3b), with the aim of advancing our comprehension of their curative value and opening more avenues for new drug discovery.

Cinnamic acid derivatives have been highly considered for their selective and least toxic molecular mechanisms, particularly through HDAC inhibition, as an option for study against cancer [11]. We intended to contribute to the ever-growing body of evidence supporting the therapeutic potential of cinnamic acid derivatives in cancer treatment by evaluating the anticancer activity of two cinnamyl sulfonamide hydroxamate compounds (derivatives 3a and 3b) as described herein [12].

Furthermore, our findings align with those from previous studies by Pontiki et al. and Reddy et al., who indicated that cinnamic acid derivatives possess anticancer properties via HDAC inhibition and the activation of apoptotic pathways [11,16]. Previously, we reported on the antioxidant and cytotoxic ability of cinnamyl sulfonamide hydroxamate compounds (derivatives 3a and 3b) [12]. In this study, we delved deeper to gain insights into the fine mechanisms involved in the anticancer pathways of these derivatives.

The impact of compounds 3a and 3b on the cell cycle of cancer cells underscores their potential to influence cancer cell growth and viability significantly. The observed variations in their effects across different cancer cell lines highlight the importance of further exploring the structure-activity relationship to optimize their therapeutic efficacy. Furthermore, understanding the molecular mechanisms underlying their cytotoxic effects is crucial for the development of more targeted cancer treatments.

Flow cytometry analysis revealed significant alterations in the cell cycle distribution of oral cavity squamous cell carcinoma (OECM 1), unique human head and neck squamous cell carcinoma (UM-SCC 6), and human tongue squamous carcinoma (HSC-3) cell lines after treatment with the synthesized compounds. Notably, a decrease in the percentage of cells in the G0/G1 phase was accompanied by an increase in the S phase, indicating a potential arrest at this phase. This suggests interference with DNA synthesis or replication processes. Moreover, statistical analysis demonstrated significant differences in cell cycle phase distribution compared to untreated control cells, affirming the impact of the compounds on cell cycle progression. These findings collectively suggest that the synthesized compounds may exert their cytotoxic effects by disrupting normal cell cycle dynamics, ultimately impairing proliferation and potentially inducing apoptosis in the tested cancer cell lines.

The investigation into p21 expression uncovered significant insights into the potential mechanisms driving cellular responses to the synthesized compounds. Analysis revealed distinct patterns of p21 expression among the OECM 1, UM-SCC 6, and HSC-3 cell lines following treatment with cinnamic acid derivatives. Particularly notable was the substantial increase in p21 expression observed in the HSC-3 cell line, indicating a role in cell cycle regulation and apoptotic processes [17].

Elevated p21 expression suggests the compounds' ability to modulate crucial cell cycle checkpoints and induce cellular stress responses, contributing to their cytotoxic effects. However, the relatively lower levels of p21 expression in the UM-SCC 6 cell line imply potential variations in the apoptotic pathways activated by the compounds across different cell types. These findings emphasize the significance of considering cell-line-specific responses and underscore the need for further elucidation of the molecular mechanisms governing variations in p21 expression. Such insights are promising for informing the development of more targeted and effective therapeutic strategies for cancer treatment.

This study exhibits several strengths, notably its systematic exploration of the therapeutic potential of cinnamyl sulfonamide hydroxamate compounds (derivatives 3a and 3b) through a comprehensive array of methodologies. The incorporation of flow cytometry analysis alongside RT-PCR assays has significantly enriched the molecular characterization, shedding light on both cell cycle progression and gene expression modulation induced by these compounds. However, it is crucial to acknowledge certain limitations, such as the absence of in vivo methods, which diminishes the translational relevance of the findings [17]. Moreover, the study's reliance on a limited set of cell lines restricts the generalizability of the results across diverse cancer types and biological contexts. Additionally, while valuable, these in vitro studies may not fully replicate the intricate physiological processes within living organisms, underscoring the necessity for further investigation using animal models and clinical settings.

Limitations

Two major limitations of the study are the potential for incomplete gene analysis, particularly in exploring additional genes related to both anti-apoptotic and proliferative pathways, and the absence of animal studies to further validate the findings. Expanding the gene analysis to encompass a broader range of pathways could provide a more comprehensive understanding of the mechanisms involved in the anticancer effects of cinnamoyl sulfonamide hydroxamate. Additionally, conducting animal studies would offer valuable insights into the compound's efficacy and safety profiles in vivo, bridging the gap between cellular mechanisms and potential clinical applications.

Furthermore, the study's reliance on anisidine and toluidine double staining to detect apoptosis presents a noteworthy limitation. While this technique distinguishes between necrotic and apoptotic cells based on morphological features, its lack of specific apoptotic markers may limit the depth of understanding regarding apoptosis induction. Therefore, caution is warranted in interpreting the study's findings on apoptosis [18]. Future research endeavors could benefit from complementing this staining method with more specific apoptosis assays, such as Annexin V staining or TUNEL assay, to offer a more comprehensive assessment. This limitation underscores the significance of employing a multifaceted approach to accurately evaluate complex cellular processes like apoptosis [19-21].

P21, also referred to as cyclin-dependent kinase inhibitor 1A (CDKN1A), is a pivotal protein governing cell cycle regulation and apoptosis. Its overexpression often serves as an indicator of apoptosis or programmed cell death. P21 exerts its function by inhibiting cyclin-dependent kinases (CDKs), crucial enzymes involved in cell cycle progression. This inhibition leads to cell cycle arrest, halting cellular division. Additionally, p21 plays a significant role in the apoptotic pathway, both dependent and independent of the p53 protein. Elevated levels of p21 expression, particularly in cancer cells, indicate the initiation of cellular responses aimed at eliminating damaged or abnormal cells. Detecting p21 overexpression, alongside other apoptotic markers, offers valuable insights into the effectiveness of anticancer treatments and the underlying mechanisms driving cell death [21].

While high levels of p21 expression may suggest the activation of specific apoptotic pathways, relying solely on p21 expression may not offer a complete understanding of apoptosis induction. Incorporating other apoptotic markers, such as cleaved caspases, Bcl-2 family proteins, and DNA fragmentation assays, can provide additional insights into the mechanisms and progression of apoptosis [22,23]. These markers help confirm apoptosis occurrence, distinguish between different apoptotic pathways, and assess the efficacy of apoptotic induction by various treatments. Therefore, analyzing a wider array of apoptotic markers alongside p21 expression would enhance the accuracy and depth of the study. This multidimensional approach would strengthen result interpretability and aid in identifying potential therapeutic targets for cancer treatment [24-26].

This study extends far beyond elucidating the therapeutic potential of cinnamyl sulfonamide hydroxamate compounds (derivatives 3a and 3b), delving into broader implications for cancer treatment and drug development. Showcasing the compounds' anti-cancer activity via HDAC inhibition and selective molecular mechanisms paves the way for novel targeted therapies across diverse cancer types. The findings emphasize the importance of exploring structurally diverse derivatives of cinnamic acid, hinting at future drug discovery endeavors [27,28]. Moreover, the study's comprehensive approach, integrating biochemical assays, flow cytometry analysis, and gene expression studies, sets a benchmark for future investigations into anti-cancer drug mechanisms. Identification of specific pathways targeted by the compounds offers insights into potential combination therapies and personalized treatment strategies. Looking ahead, these findings promise advancements in precision medicine in oncology, ushering in more effective and less toxic cancer treatments. Future research directions may include further preclinical testing, optimization of compound formulations, and clinical trials, culminating in tangible benefits for cancer patients.

The present research underscores the robust anti-cancer properties of cinnamic acid derivatives, hinting at their potential for designing novel anti-cancer agents. However, thorough examinations are still warranted to unveil the complete mechanistic actions in treating various cancer forms and optimizing efficacy against diverse cancer types.

## Conclusions

Cinnamyl sulfonamide hydroxamate compounds, particularly derivatives 3a and 3b, have emerged as notable contenders in the realm of cancer treatment due to their distinctive ability to selectively inhibit HDACs while concurrently stimulating apoptotic pathways. This dual action holds significant promise for combating cancer, particularly in the context of oral cancer, where these compounds have demonstrated marked cytotoxicity across various cell lines. Notably, they induce cell cycle arrest and diminish cell viability, showcasing their potential as effective anticancer agents.

Moreover, the exploration of the molecular mechanisms behind the actions of these compounds has unveiled crucial insights, providing a foundation for the development of more potent and less toxic therapies for cancer. By delving into the intricate pathways they target, researchers have illuminated new avenues for targeted therapies, potentially revolutionizing the treatment landscape for OSCC and ADC, among other cancer types.

In essence, this research not only highlights the pharmacological prowess of cinnamic acid derivatives but also underscores the tantalizing prospect of tailored therapies that could significantly improve outcomes for patients battling various forms of cancer.
